# Zeylenone Induces Mitochondrial Apoptosis and Inhibits Migration and Invasion in Gastric Cancer

**DOI:** 10.3390/molecules23092149

**Published:** 2018-08-27

**Authors:** Shuxian Yang, Yonghong Liao, Liyong Li, Xudong Xu, Li Cao

**Affiliations:** Institute of Medicinal Plant Development, Chinese Academy of Medical Sciences & Peking Union Medical College, Beijing 100193, China; Yangshuxianlove@163.com (S.Y.); yhliao@implad.ac.cn (Y.L.); lly0916@sina.com (L.L.); xdxu@implad.ac.cn (X.X.)

**Keywords:** Zeylenone, gastric cancer, invasion, migration, apoptosis

## Abstract

The mortality of gastric cancer (GC) is increasing due to its high rates of recurrence and metastasis. Zeylenone (Zey), a type of naturally occurring cyclohexene oxide, was demonstrated to be effective in cancer patients. The aim of this study is to explore the anti-cancer effect of Zey against gastric cancer both in vitro and in vivo, as well as the underlying mechanisms. We found that Zey inhibited gastric tumor growth, as demonstrated by in vitro gastric cancer cell lines and in a human gastric cancer xenograft mouse model. Furthermore, Zey induced substantial apoptosis through a mitochondrial apoptotic pathway, involving mitochondrial transmembrane potential loss, caspase-3 activation, anti-apoptotic protein downregulation, and pro-apoptotic protein upregulation. Notably, we revealed for the first time that Zey suppressed invasion and migration by wound healing and transwell chamber assays. Through Western blotting, we further explored the potential mechanism of Zey’s anti-cancer activity. We found that Zey downregulated the expression of matrix metalloproteinase 2/9 (MMP 2/9) and inhibited the phosphorylation of AKT and ERK. In short, Zey, which induced mitochondrial apoptosis and inhibited proliferation, migration, and invasion, may be developed as a novel drug for the treatment of gastric cancer.

## 1. Introduction

Gastric cancer (GC) represents a health threat as the fourth most common cancer and the second leading cause of cancer death worldwide [1,2]. Studies showed that more than 950,000 new diagnoses are made every year, and an estimated 720,000 patients died from gastric cancer in 2012 [3]. Even with the rapid improvement of healthcare and detection [4], gastric cancer is still a nightmare, and the relative five-year survival rates for GC are only about 20% in most areas of the world [5]. Thus, the burden of gastric cancer remains high, especially in Asia, Latin America, and central and eastern Europe [6]. Furthermore, gastric cancer is characterized by uncontrolled cell proliferation and metastasis [2]. Therefore, most patients do not die due to the primary cancer, but from metastatic cancer [7]. However, research into the field of metastasis, compared with other key events such as proliferation, is lacking. Hence, identifying new drugs with anti-metastasis and anti-proliferation characteristics is now the focus in GC treatment.

Naturally occurring bioactive phytochemicals with low toxicity emerged as promising options for the development of effective alternatives for conventional treatments. Zeylenone (Zey) [8], isolated from ethanol extracts of the leaves of *Uvaria grandiflora* Roxb., is a cyclohexene oxide [9], which exhibits anti-cancer activity and is regarded as a soft drug. In 2010–2012, we successfully attained mPEG-PLGA-loaded Zeylenone nanomicelles, which improved the solubility and stability of Zey and achieved sustained release. The drug-loaded micelles were also characterized in terms of drug encapsulation, dynamic size, zeta potential, drug stability, and release, involving not just in vitro release assays, but also in vivo pharmacokinetic studies [8]. In addition, previous studies by our group indicated that Zey displayed strong cytotoxic activity against acute lymphoblastic leukemia cells [10] and cervical cancer cells [11] in a dose-dependent manner, indicating its strong antitumor activity. However, to the best of our knowledge, the effect of Zey on gastric cancer is yet to be studied in detail, especially the effect of Zey on gastric cancer invasion and migration.

In view of the above, there is an interest in studying the potential effects and mechanisms of Zey against GC. In this study, we found that Zey inhibited gastric tumor growth, as demonstrated by in vitro gastric cancer cell lines and in a human gastric cancer xenograft mouse model. Our further studies revealed that Zey induced substantial apoptosis of GC cells, which was associated with the mitochondrial apoptotic pathway. In addition, Zey also suppressed the invasion and migration of gastric cancer cells by wound healing and transwell chamber assays.

The activation of the AKT and ERK pathway is necessary for tumor initiation and progression, including cell growth, metastasis, and resistance to chemotherapy [12]; thus, these pathways are worth studying [13]. ERK is an important member of the MAPK family, which plays a central role in regulating the expressions of matrix metalloproteinases (MMPs) [14]. MMPs, a family of zinc-dependent neutral endopeptidases, are involved in the metastasis of cancer because of their ability to hydrolyze various extracellular matrix (ECM) components [15]. Of note, MMP-2 and MMP-9 can degrade most ECM components, accelerating metastasis [16]. Through Western blotting, we further explored the potential mechanism of Zey’s anti-cancer activity. We found that Zey suppressed the metastasis of gastric cancer cells by decreasing protein levels of MMP-2 and MMP-9 and inhibiting the phosphorylation of AKT, ERK, and mTOR. According to the above experimental results, we conclude that Zey may be developed into a novel therapeutic agent for gastric cancer treatment due to its strong ability to inhibit migration and invasion and to induce apoptosis.

## 2. Results

### 2.1. Zey Inhibits Gastric Cancer Cell Proliferation 

**Figure 1:**
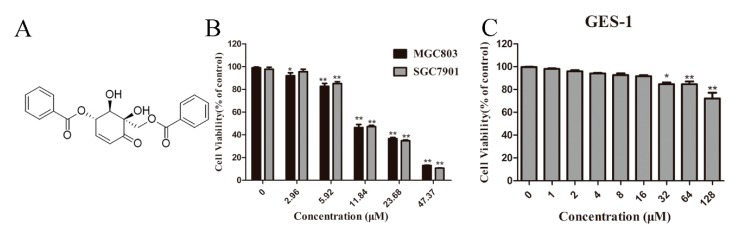

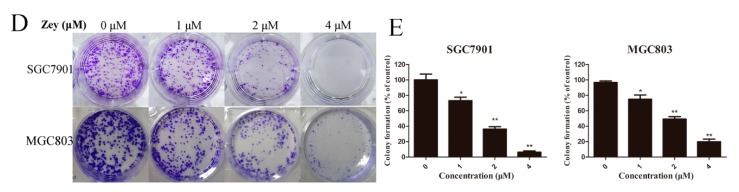
Zeylenone (Zey) effectively suppresses cell viability and colony formation in SGC7901 and MGC803 cells. (**A**) Chemical structure of Zey. (**B**) Zey suppresses the viability of SGC7901 and MGC803 cells determined in an MTT assay. Cells were treated with Zey (0, 2.96, 5.92, 11.84, 23.68, or 47.37 μM) for 24 h. Data are expressed as means ± SD of three distinct experiments. The cell viability of the control (DMSO only) is indicated as 100%. * *p* < 0.05 and ** *p* < 0.01 vs. control cells. (**C**) Zey suppresses the viability of normal gastric epithelial (GES-1) cells determined in an MTT assay. GES-1 cells were treated with Zey (0, 1, 2, 4, 8, 16, 32, 64, or 128 μM) for 24 h. Data are expressed as means ± SD of three distinct experiments. * *p* < 0.05 and ** *p* < 0.01 vs. control cells. (**D**) Representative images of colonies after SGC7901 and MGC803 cells were treated with Zey for 12 days. (**E**) Statistical analysis of colony numbers from three independent experiments. * *p* < 0.05 and ** *p* < 0.01 vs. control cells.

The chemical structure of Zey is shown in Figure 1A. An MTT assay and colony formation assay were performed to determine the anti-proliferative effect of Zey on SGC7901 and MGC803 cells. As shown in Figure 1B,C, Zey treatments reduced the cell viability of the SGC7901 and MGC803 cells in a dose-dependent manner without severe toxicity to normal gastric epithelial cells (GES-1). Similar results from a colony formation assay confirmed that Zey-treated groups exhibited smaller and fewer colonies compared to untreated cells (Figure 1D,E). However, low concentrations of Zey (<3 μM) did not significantly inhibit cell viability. IC_50_ values of Zey were detected as 13.21 μM for SGC7901 cells and 13.42 μM for MGC803 cells. These data together indicated that Zey has anti-proliferation activity in gastric cancer cells.

### 2.2. Zey Induces Gastric Cancer Cell Apoptosis 

**Figure 2:**
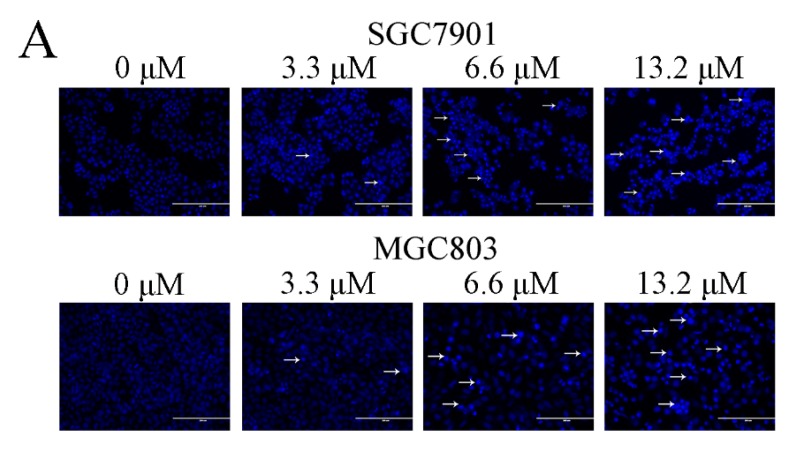

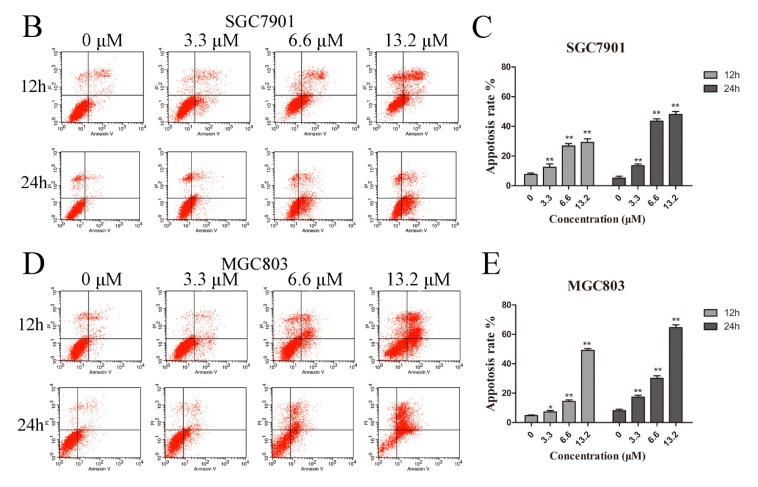
Zey induces apoptosis in SGC7901 and MGC803 cells. (**A**) Morphological changes of apoptosis were detected by Hoechst 33258 staining. Images are shown under a fluorescence microscope with a magnification of 200×. (**B**,**C**) Apoptosis of SGC7901 cells detected by the Annexin V-FITC/PI staining test and the ratio of apoptotic cells. The data represented the means ± SD for triplicate determinations. * *p* < 0.05 and ** *p* < 0.01 vs. control cells. (**D**,**E**) Apoptosis of MGC803 cells detected by the Annexin V-FITC/PI staining test and the ratio of apoptotic cells, including early apoptotic cells (lower right quadrant) and late apoptotic cells (upper right quadrant). The data represented the means ± SD for triplicate determinations. * *p* < 0.05 and ** *p* < 0.01 vs. control cells.

We further evaluated whether Zey inhibited cell proliferation by inducing cancer cell apoptosis. Firstly, Hoechst 33258 staining was used to study the morphological changes of GC cells. Based on IC_50_ values, we chose 3.3, 6.6, and 13.2 μM as treatments for GC cells. After Hoechst 33258 staining, an increased number of cells with bright nuclear condensation or fragmented nuclei, which was regarded as characteristic of cell apoptosis, was observed after Zey treatment, while control cells exhibited round nuclei and the chromatin were well distributed (Figure 2A).

Next, we further assessed apoptosis using annexin V-FITC/PI apoptosis staining. After treatment with Zey for 12 h, the apoptosis rate significantly increased in a dose-dependent manner in SGC7901 and MGC803 cells (Figure 2B–E). With the treatment time of Zey extended to 24 h, at 13.2 μM, the apoptosis rates of SGC7901 and MGC803 cells reached up to 48.21% and 64.58% (*p* < 0.01), respectively. Thus, Zey induced gastric carcinoma cell apoptosis in a dose- and time-dependent manner.

### 2.3. Zey Induces Apoptosis in Gastric Cancer Cells via the Mitochondrial Apoptosis Pathway

To understand the underlying mechanism via which Zey induces apoptosis in GC cells, we investigated changes in the mitochondrial membrane potential and the level of corresponding proteins involved in apoptosis.

**Figure 3:**
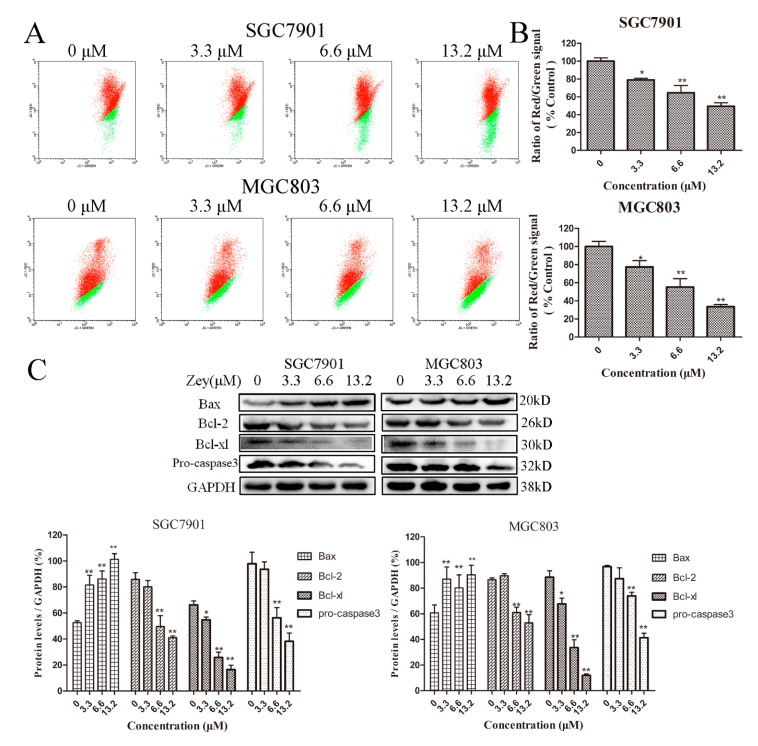
Zey induces apoptosis of SGC7901 and MGC803 cells through the initiation of the mitochondrial pathway. (**A**) Zey induced a loss of mitochondrial membrane potential in SGC7901 and MGC803 cells. Cells were treated with increasing concentrations of Zey for 24 h, and mitochondrial membrane potential was analyzed by flow cytometry after cells were stained with JC-1. (**B**) Quantitative analysis of the ratio of red to green fluorescence. The data represented the means ± SD for triplicate determinations. * *p* < 0.05 and ** *p* < 0.01 vs. control cells. (**C**) Apoptosis-related protein expression was detected by Western blot analysis in SGC7901 and MGC803 cells. Cells were incubated with Zey (0, 3.3, 6.6, or 13.2 μM) for 24 h, and whole-cell lysates were analyzed. The data represented the means ± SD for triplicate determinations. * *p* < 0.05 and ** *p* < 0.01 vs. control cells.

Initially, cells treated with or without Zey were stained with JC-1, and the changes in mitochondrial membrane potential were analyzed by flow cytometry. As shown in Figure 3A,B, when the concentration of Zey was 3.3 μM, the effect was obvious neither in SGC7901 cells nor in MGC803 cells. However, Zey treatment at 13.2 μM induced a loss of mitochondrial membrane potential (*p* < 0.01). 

Next, we further measured the effect of Zey on proteins related to apoptosis with Western blotting (Figure 3C). Zey treatment induced a significant decline in anti-apoptotic proteins Bcl-xl and Bcl-2 while significantly increasing the pro-apoptotic protein Bax. Consistently, Zey treatment markedly decreased the levels of pro-caspase-3, indicating that Zey treatment induced gastric cancer cell apoptosis via caspase-3 activation and the involvement of anti-apoptotic proteins Bcl-xl and Bcl-2 and pro-apoptotic protein Bax.

### 2.4. Zey Inhibits the Migration and Invasion of Gastric Cancer Cells

**Figure 4:**
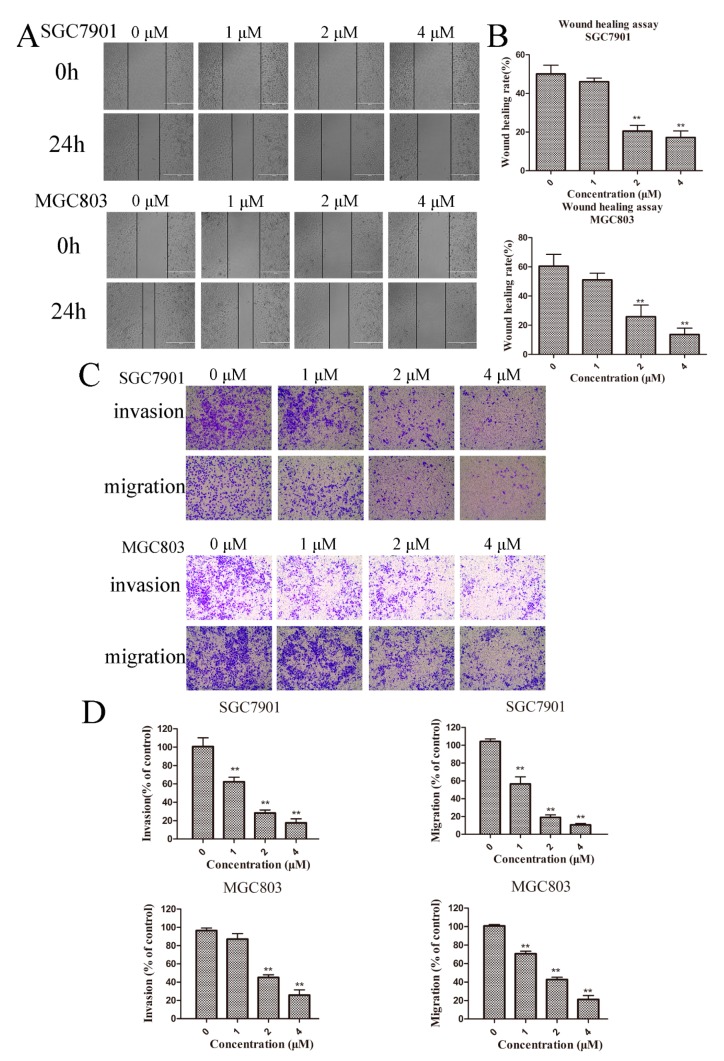
Zey inhibits the migration and invasion in SGC7901 and MGC803 cells. (**A**) Wound-healing assay in SGC7901 and MGC803 cells. After making a linear wound, cells were incubated with different concentrations of Zey (0, 1, 2, or 4 μM) in serum-free RPMI-1640 for an additional 24 h. Wound healing was photographed at 0 and 24 h using a microscope with a magnification of 100×. (**B**) Quantitative analysis of the ratio of wound healing. The data represented the means ± SD for triplicate determinations. * *p* < 0.05 and ** *p* < 0.01 vs. control cells. (**C**) Invasion and migration assay in SGC7901 and MGC803 cells. Images are shown under a light microscope with a magnification of 100×. (**D**) Quantitative analysis of the ratio of invasion/migration in SGC7901 and MGC803 cells. The data represented the means ± SD of three distinct experiments. The control cells were regarded as 100% in the invasion/migration index calculation. * *p* < 0.05 and ** *p* < 0.01 vs. control cells.

Previous studies demonstrated that cancer metastasis is highly related to cellular motility and degradation of the ECM [17]. We first examined the effect of Zey on motility in gastric cancer cells using a wound-healing assay. According to the cell viability data (Figure 1B), we chose Zey concentrations at 1, 2, and 4 μΜ (with a cell viability rate >93%). As shown in Figure 4A,B, the wound-healing ability in SGC7901 cells decreased gradually in a dose-dependent manner with raised concentrations of Zey, consistent with the results in MGC803 cells. Of note, when the concentration of Zey increased to 2 μM and 4 μM, the wound-healing rates of SGC7901 and MGC803 cells were reduced to 20.50% and 25.82% at 2 μM and to 17.94% and 13.62% at 4 μM, respectively. At a low concentration (1 μM), the effect was not as significant (*p* > 0.05).

Next, the effect of Zey on migration and invasion was also further determined with a transwell chamber assay. After treatment with Zey (0, 1, 2, or 4 μM) for 24 h, the number of Zey-treated SGC7901 cells that invaded the lower chamber was significantly less than the control group (*p* < 0.01, Figure 4C,D). At 4 μM, the invasion and migration rates of SGC7901 were 17.6% and 10.5%, respectively. Then, the same effects were also discovered in MGC803 cells, with percentages of 25.7% and 21.1%, respectively. It can be seen that the effect of Zey on inhibiting invasion and migration in MGC803 cells was not as obvious as in SGC7901 cells. Collectively, these findings showed that Zey could inhibit migration and invasion of GC cells in a dose-dependent manner.

### 2.5. Zey Attenuates AKT/MMP2/MMP9 and ERK Signaling Pathways in Gastric Cancer Cells

**Figure 5:**
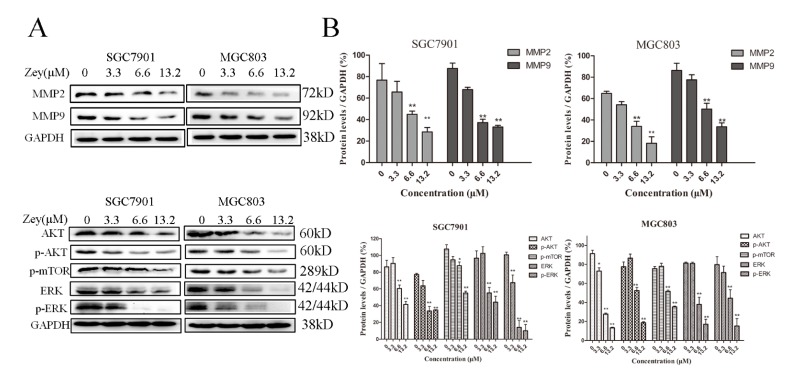
Zey attenuates AKT/matrix metalloproteinase 2 (MMP2)/MMP9, and ERK pathways in SGC7901 and MGC803 cells. (**A**) Immunoblot analyses of AKT, phosphorylated (p)-AKT, MMP-2, MMP-9, p-mTOR, ERK, and p-ERK in Zey-treated SGC7901 and MGC803 cells. (**B**) Quantitative analysis of protein levels. GAPDH was used to confirm equal protein loading. The data represented the means ± SD for triplicate determinations. * *p* < 0.05 and ** *p* < 0.01 vs. control cells.

To further understand the experimental results mentioned above, we continued exploring the possible mechanisms with Western blotting, particularly with MMP-2 and MMP-9 [18]. As shown in Figure 5A,B, Zey (6.6 and 13.2 μM) treatment significantly reduced the expressions of MMP-2 and MMP-9 compared with the control group (*p* < 0.01). With the concentration of Zey increased to 6.6 μM and 13.2 μM, the inhibitory effect on the activity of MMP-2 and MMP-9 appeared to be more obvious. Activation of AKT accelerated cancer progression and distant metastasis. Our result showed that Zey blocked the phosphorylation of AKT and ERK in SGC7901 and MGC803 cells, and the result was consistent between SGC7901 and MGC803 cells. Collectively, these data indicated that the antitumor effect of Zey on SGC7901 and MGC803 cells is tightly correlated with the AKT/MMP2/MMP9 and ERK signaling pathways.

### 2.6. Zey Suppresses Tumor Growth in a Mouse Xenograft Model

**Figure 6:**
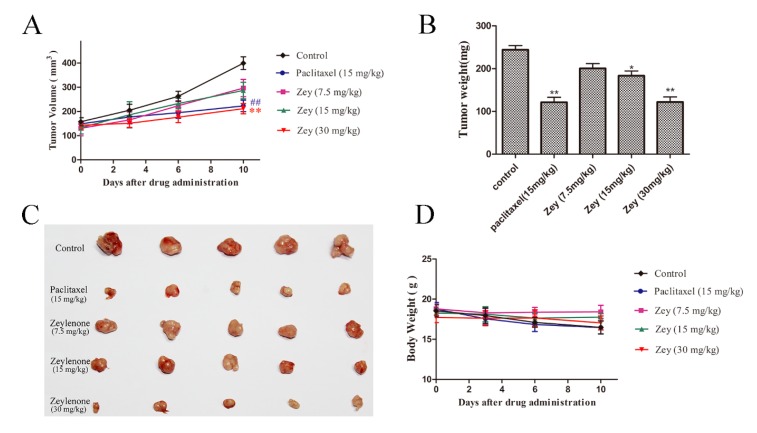
Zey inhibits tumor growth in nude BALB/c mice bearing BGC823 xenografts. BGC823 cells were transplanted subcutaneously to BALB/c nude mice and administered Zey (7.5, 15, and 30 mg/kg), paclitaxel (15 mg/kg), and saline (as the negative control) for 10 days. (**A**) Tumor volume was recorded every two days. Mean ± SD (n = 5). ^##^ *p* < 0.01 and ** *p* < 0.01 vs. control group. (**B**) Tumor weight of each group. On day 10 after inoculation, the mice were sacrificed, and the tumor tissues were isolated, weighed, and summarized. Mean ± SD (n = 5). * *p* < 0.05 and ** *p* < 0.01 vs. control group. (**C**) Photographs of tumors in each group. (**D**) Body weight was measured every two days. Mean ± SD (n = 5).

To further validate the antitumor activity of Zey in vivo, we developed a xenograft model of GC, and tested the effects of Zey on tumor growth. After treatment with Zey for 10 days, the tumor growth in the Zey treatment groups was significantly slower compared with the control groups (Figure 6A). In detail, no significant difference in the tumor volume of the different groups was observed at the beginning of treatment. At the end of treatment, as expected, the tumor volume was smaller in the Zey (30 mg/kg) groups compared with the control groups (*p* < 0.01), as was paclitaxel. Alternatively, the isolated tumor weight was also remarkably reduced in the Zey-treated group (30 mg/kg) than in the control group (Figure 6B,C). Simultaneously, the tumor inhibition rates of Zey were 47.95% (30 mg/kg) and 48.11% (15 mg/kg) in the paclitaxel group compared to the control group (Figure 6B). Moreover, the mouse body weights in the Zey-treated groups slightly decreased, but not obviously (*p* > 0.05), indicating that Zey was not significantly toxic (Figure 6D). Together, these data indicated that Zey could effectively inhibit gastric tumor growth in vivo. In the in vivo experiments reported here, the Zey was mPEG-PLGA-loaded Zeylenone nanomicelles.

## 3. Discussion

In the present study, we attempted to identify novel functions of Zey and proposed that Zey may be a potent suppressor of gastric cancer cells. On the other hand, the therapeutic efficacy of Zey against gastric cancer and the potential mechanism remained unclear. Therefore, we evaluated the anti-cancer effects of Zey both in vitro and in a mouse xenograft model and further investigated the underlying mechanisms.

Gastric cancer is one of the most common malignant diseases and ranks second in mortality among all cancers worldwide [19,20]. Although the detection of early gastric cancer improved, long-term survival remains poor. Firstly, Zey showed strong antitumor activity against SGC7901 and MGC803 cells, and therefore, we performed a further and deeper study of Zey in gastric cancer.

Previous studies showed that apoptosis induction played an important role in the inhibition of cancer cells [21]. Relevant studies confirmed that the imbalance between pro-apoptotic proteins, such as Bax, versus anti-apoptotic factors, such as Bcl-2 and Bcl-xl, can result in decreased mitochondrial membrane integrity, leading to the release of cytochrome C and AIF from mitochondria to the cytoplasm. The released cytochrome C then activates caspase, which finally leads to apoptosis [22]. Consistently, in this study, we found that Zey treatment induced apoptosis by dose-dependently decreasing the levels of anti-apoptotic proteins, including Bcl-2 and Bcl-xl, and increasing the expression of the pro-apoptotic protein Bax. Moreover, Zey led to cell apoptosis with the loss of mitochondrial membrane potential, and apoptotic morphological alterations were also observed in GC cells. Taken together, increased apoptosis might be responsible for cancer growth inhibition by Zey [11].

Notably, metastasis, as one of the most important factors of cancer, limits tumor prognosis and increases cancer-related mortality. Therefore, we first found that Zey suppressed both migration and invasion in SGCC7901 and MGC803 cells. To explore the mechanism, we examined the expression of MMPs. Previous studies showed that MMP-2 [23] and MMP-9 [24] were overexpressed in gastric cancer, which accelerated the invasion of tumor cells in vitro and in vivo. In other words, the activation of MMPs led to the spread of tumor cells from their original locations. In our study, we confirmed that Zey treatment markedly reduced MMP-2/9 expression in SGC7901 and MGC803 cells, which implied that Zey, as an inhibitor of MMP expression [25], may be developed into an early treatment to prevent cancer metastasis [26].

In addition, we also focused on the ERK and AKT pathways because they serve critical roles in regulating both the expression of MMPs and cell apoptosis [27]. High expression levels of AKT and phosphorylated (p)-AKT were observed in 74% and 78% of gastric tumors, respectively [28]. Additionally, aberrant AKT activation plays a substantial role in invasion and metastasis. Likewise, ERK is an important member of the MAPK family, which plays a central role in regulating the expressions of MMPs [29]. Based on the above results, we tested the related proteins using Western blot assays. As expected, Zey decreased the expressions of AKT, p-AKT, p-mTOR, ERK, and p-ERK, which may partly account for the proliferative inhibition and suppressive migration observed in the GC cells. However, to some extent, a limitation of our study is that we did not identify the inhibitory target of Zey in these pathways. Therefore, further studies are needed.

To determine whether Zey inhibits tumor growth in vivo, a human gastric cancer xenograft mouse model was used. We found that Zey administered at 30 mg/kg slowed the growth of BGC823 tumors, resulting in a significantly decreased tumor weight on day 10 in the Zey-treated group compared to control mice (*p* < 0.01). Moreover, the treatments of Zey at 30 mg/kg every two days and paclitaxel at 15 mg/kg every two days caused similar proliferation inhibition profiles (inhibition rate at 47.95% and 48.11%, respectively). This was evidence that Zey may be a cytotoxic drug. These findings in vivo were certainly consistent with the observations in vitro and further indicate that Zey may be a potential candidate as an active agent against gastric cancer.

Since Zeylenone was discovered in 1997, we completed pre-clinical studies, including pharmacochemistry [9], structural-activity relationship (SAR) studies [30], drug dosage forms [8], and quantitative analysis of differential protein expression in cervical cancer cells [11]. In the experiments reported here, we mainly discussed the pharmacodynamics and potential mechanism of Zey in gastric cancer. Firstly, we confirmed that Zey inhibited the proliferation of gastric cancer cells in vitro. Then, we went back to the tumor-bearing mice to verify the inhibition of tumor growth in vivo. Finally, we returned to the experiments in vitro again to explore Zey’s anti-cancer activity and underlying mechanism. Based on our current preclinical findings, Zey shows promise for development as a novel therapeutic agent in gastric cancer treatment [31]. In the future, when we complete the safety evaluation and pharmacodynamics study, we can go from non-clinical research to clinical research, and we hope that Zey can be used in humans someday.

In summary, our study demonstrated potential effects of Zey on gastric carcinoma using diverse gastric cell lines and a human gastric cancer xenograft mouse model. It may be possible in the future to develop a therapeutic strategy or novel drug for patients with gastric cancer.

## 4. Materials and Methods 

### 4.1. Materials

Preparations of Zeylenone (Zey) and mPEG-PLGA-loaded Zeylenone nanomicelles were described previously [8]. Zey used for the in vitro studies was stored as 150 mM solutions in DMSO at −20 °C and further diluted to desired working concentrations before use (DMSO concentration <1%). The mPEG-PLGA-loaded Zeylenone nanomicelles used for the in vivo studies were stored in a dry container at room temperature. 

RPMI-1640, DMEM, and fetal bovine serum (FBS) were purchased from Corning Inc. (Corning, NY, USA). MTT, Hoechst 33258, and fluorescent dye JC-1 were purchased from Sigma Aldrich (St. Louis, MO, USA). Antibodies against Bcl-2, Bcl-xl, MMP-9, and MMP-2 were purchased from Santa Cruz Biotechnology (Santa Cruz, CA, USA). Antibodies against Bax, p-AKT, AKT, p-mTOR, pro-caspase3, p-ERK, ERK, and GAPDH, as well as all secondary antibodies, were purchased from Cell Signaling Technology (Danvers, MA, USA). An Annexin V-FITC/PI kit and Matrigel matrix were obtained from BD Biosciences (San Jose, CA, USA). 

### 4.2. Cell Lines and Cell Culture

Human gastric carcinoma cell lines, SGC7901, MGC803, and BGC823, and the human normal gastric epithelial cell line, GES-1, were all obtained from the Chinese Academy of Medical Sciences Basic Medicine Cell Center (Beijing, China). SGC7901, MGC803, and BGC823 cells were maintained in RPMI-1640 media containing 10% FBS and 1% penicillin/streptomycin in a 37 °C humidified incubator with 5% CO_2_. GES-1 cells were cultured in DMEM media under the same conditions.

### 4.3. Ethics Statement and Animals

All animal experiments and care were performed in accordance with the National Institutes of Health regulations for the care and use of animals in research. All mouse protocols were approved by the Animal Ethics Committee at the Institute of Medicinal Plant Development, Chinese Academy of Medical Sciences (No. SLXD-2017051634) and were in compliance with the Chinese Association for Laboratory Animal Sciences guidelines.

Female BALB/c nude mice (14–16 g) were purchased from SPF Biotechnology Co., Ltd. (Certificate no. SCXK-2016-0002, Beijing, China) and were maintained on a 12-h light/dark cycle with controlled humidity (50–70%) and temperature (20–24 °C). 

### 4.4. Cell Viability Assay by MTT

Cells cultured in 96-well plates at a density of 5 × 10^3^ cells/well were treated with various concentrations of Zey for 24 h, and cell viability was measured with an MTT assay, as previously described [32].

### 4.5. Colony Formation Assay

Cells (400 cells/well, six-well plates) were treated with Zey (0, 1, 2, or 4 μΜ) and were incubated in a 37 °C humidified atmosphere with 5% CO_2_ for 12 days. Cell aggregates containing 50 or more cells were considered colonies. The colonies were fixed with methanol, stained with 0.1% Coomassie blue, and then counted under a microscope.

### 4.6. Hoechst 33258 Staining

SGC7901 and MGC803 cells were treated with different concentrations of Zey for 24 h. After washing with phosphate-buffered saline (PBS), the cells were stained with Hoechst 33258 (10 μg/mL) for 20 min. Nuclear morphology changes were observed and then captured using a fluorescence microscope (Olympus, Tokyo, Japan).

### 4.7. Flow Cytometric Analysis of Apoptosis

Cells cultured in six-well plates were treated with Zey (SGC7901 and MGC803: 0, 3.3, 6.6, and 13.2 μM) for 12 h and 24 h, respectively. After harvesting and washing twice with PBS, the cells were stained with annexin-V-FITC and PI according to the manufacturer’s directions. The stained cells (10^4^ cells) were then analyzed immediately using an FACS Calibur flow cytometer (Becton Dickinson, CA, USA), and the results were expressed as a percentage of living (AnnV−, PI−), early apoptotic (AnnV+, PI−), and late apoptotic/dead cells (AnnV+, PI+). Apoptotic rates were reported as the percentage of apoptotic cells (including early apoptotic cells and late apoptotic cells) among total cells. 

### 4.8. JC-1 for Mitochondrial Transmembrane Potential Study

To investigate whether apoptosis was related to the mitochondrial apoptosis pathway, we measured the change of mitochondrial transmembrane potential using JC-1 [33]. Cells were treated with different concentrations of Zey (SGC7901 and MGC803: 0, 3.3, 6.6, or 13.2 μM) for 24 h. The cells were then collected and stained with 10 μg/mL JC-1 for 30 min in the dark at 37 °C. After washing twice with PBS, the cells were analyzed by flow cytometry. The highly negative membrane potential in mitochondria produces JC-1 red fluorescence. Loss of mitochondrial transmembrane potential results in green fluorescence and loss of the red fluorescence.

### 4.9. Wound-Healing Assays

Wound-healing assays were performed to investigate migration under Zey treatment. When cell density in 24-well plates was approximately 90%, a sterile 10-μL pipette tip was used to make a linear wound. Cells were washed to remove superfluous floating cells and debris, then co-incubated with different concentrations of Zey (0, 1, 2, or 4 μΜ, with a cell viability rate >93%) in serum-free RPMI-1640 for an additional 24 h. Wound healing was photographed at 0 and 24 h using a microscope. The width of the wound was measured by ImageJ software (National Institutes of Health, Bethesda, MD, USA). The wound-healing ratio (%) = (wound area at 0 h − wound area at 24 h)/wound area at 0 h × 100%.

### 4.10. Invasion and Migration Assay

For invasion and migration assays, 8.0-μm-pore-size transwell chambers (Costar, Corning Inc., Corning, NY, USA) were used [34]. Transwell chambers were coated with Matrigel (dilution 1:5) for the invasion assays. After solidification, the cells (4 × 10^4^) treated with Zey were seeded on the upper transwell chamber insert in 24-well plates and cultured in serum-free RPMI-1640 medium. Meanwhile, 500 μL of RPMI-1640 medium containing 10% FBS was added to the 24-well plates. After 24 h, non-invading cells on the upper surface of the membranes were wiped out by cotton swab and those on the underside were stained with 0.1% crystal violet and counted under a light microscope with a magnification of 100×. For each replicate, the cells in 10 randomly selected fields were determined, and the counts were averaged. The invasion rate (%) = the number of migrated cells at 1, 2, or 4 μM/the number of migrated cells at 0 μM × 100%. Similar methods were performed for migration assays, except Matrigel was not used.

### 4.11. Western Blot Analyses

Cells were exposed to Zey (SGC7901 and MGC803: 0, 3.3, 6.6, or 13.2 μM) for 24 h. The detailed steps for electrophoresis, transfer, and immunoblotting were described previously [35]. Antibodies used in this study were mentioned in the Materials and Methods section. All experiments were performed in triplicate, and levels of protein expression were quantified using the Image J software.

### 4.12. Tumor Xenograft Study

A total of 2 × 10^7^ BGC823 human gastric cancer cells were inoculated subcutaneously on the right flanks of mice. The mouse body weight and tumor size were measured every two days. The tumor volume (V) was calculated using the formula V = (L × W^2^)/2, where L is the largest diameter and W is the diameter perpendicular to width. When tumors reached 100–150 mm^3^, the mice were randomized to five groups (n = 5) and treated with vehicle (saline), mPEG-PLGA-loaded Zeylenone micelles (containing Zeylenone 7.5, 15, or 30 mg/kg, once every two days, tail vein injection), and paclitaxel (15 mg/kg, once every two days, tail vein injection) for a total of 10 days. Subsequently, the mice were then euthanized, and the tumors were excised, weighed, fixed, and stored.

### 4.13. Statistical Analysis 

All experiments were performed in triplicate, and data were presented as the means ± SD. The GraphPad Prism 6.0 software (GraphPad Software, La Jolla, CA, USA) was used for statistical analysis. The statistical significance of group differences was analyzed using one-way (ANOVA) and a Tukey’s post hoc test (*t*-test), and *p* < 0.05 was considered to be statistically significant.
